# Specific and Sensitive Visual Proviral DNA Detection of Major Pathogenic Avian Leukosis Virus Subgroups Using CRISPR-Associated Nuclease Cas13a

**DOI:** 10.3390/v16071168

**Published:** 2024-07-20

**Authors:** Qingqing Xu, Yaoyao Zhang, Yashar Sadigh, Na Tang, Jiaqian Chai, Ziqiang Cheng, Yulong Gao, Aijian Qin, Zhiqiang Shen, Yongxiu Yao, Venugopal Nair

**Affiliations:** 1 The Pirbright Institute and UK-China Centre of Excellence for Research on Avian Diseases, Pirbright, Guildford, Surrey GU24 ONF, UK; zjzlxqq@126.com (Q.X.); yaoyao.zhang@pirbright.ac.uk (Y.Z.); Yashar.sadigh@coventry.ac.uk (Y.S.); 2 UK-China Centre of Excellence for Research on Avian Diseases, Shandong Binzhou Animal Science and Veterinary Medicine Academy, Binzhou 256600, China; tangna0543@163.com (N.T.); bzshenzq@163.com (Z.S.); 3 Sino-UK Laboratory for Poultry Disease Research, Shandong Binzhou Animal Science and Veterinary Medicine Academy, Binzhou 256600, China; 4 College of Veterinary Medicine, Shandong Agricultural University, Tai’an 271018, China; jqchai@sdau.edu.cn (J.C.); czqsd@126.com (Z.C.); 5 State Key Laboratory of Veterinary Biotechnology, Division of Avian Infectious Diseases, Harbin Veterinary Research Institute, The Chinese Academy of Agricultural Sciences, Harbin 150008, China; gaoyulong@caas.cn; 6 Ministry of Education Key Lab for Avian Preventive Medicine, Yangzhou University, Yangzhou 225109, China; aijian@yzu.edu.cn; 7 The Jenner Institute Laboratories, University of Oxford, Oxford OX3 7DQ, UK; 8 Department of Biology, University of Oxford, Oxford OX1 3RB, UK

**Keywords:** avian leukosis viruses, CRISPR-Cas13a diagnosis, RPA, Lateral flow detection, ALV detection

## Abstract

Avian leukosis viruses (ALVs) include a group of avian retroviruses primarily associated with neoplastic diseases in poultry, commonly referred to as avian leukosis. Belonging to different subgroups based on their envelope properties, ALV subgroups A, B, and J (ALV-A, ALV-B, and ALV-J) are the most widespread in poultry populations. Early identification and removal of virus-shedding birds from infected flocks are essential for the ALVs’ eradication. Therefore, the development of rapid, accurate, simple-to-use, and cost effective on-site diagnostic methods for the detection of ALV subgroups is very important. Cas13a, an RNA-guided RNA endonuclease that cleaves target single-stranded RNA, also exhibits non-specific endonuclease activity on any bystander RNA in close proximity. The distinct trans-cleavage activity of Cas13 has been exploited in the molecular diagnosis of multiple pathogens including several viruses. Here, we describe the development and application of a highly sensitive Cas13a-based molecular test for the specific detection of proviral DNA of ALV-A, B, and J subgroups. Prokaryotically expressed LwaCas13a, purified through ion exchange and size-exclusion chromatography, was combined with recombinase polymerase amplification (RPA) and T7 transcription to establish the SHERLOCK (specific high-sensitivity enzymatic reporter unlocking) molecular detection system for the detection of proviral DNA of ALV-A/B/J subgroups. This novel method that needs less sample input with a short turnaround time is based on isothermal detection at 37 °C with a color-based lateral flow readout. The detection limit of the assay for ALV-A/B/J subgroups was 50 copies with no cross reactivity with ALV-C/D/E subgroups and other avian oncogenic viruses such as reticuloendotheliosis virus (REV) and Marek’s disease virus (MDV). The development and evaluation of a highly sensitive and specific visual method of detection of ALV-A/B/J nucleic acids using CRISPR-Cas13a described here will help in ALV detection in eradication programs.

## 1. Introduction

Avian leukosis virus (ALV) is a major group of avian pathogens associated with severe neoplastic disease affecting multiple cell types commonly referred to as avian leukosis. The prevalence of multiple ALV envelope subgroups together with widespread diversity and recombination make the detection of ALV very challenging [1]. Among the commercial poultry populations, ALV subgroups A, B, and J (ALV-A, ALV-B, and ALV-J) are the most common ALVs. Viruses belonging to ALV-A and ALV-B, widespread in some countries, are primarily associated with lymphoid leukosis (LL) and sarcomas. While diseases caused by ALV-C and ALV-D are less commonly reported, ALV-E is the ubiquitous endogenous retrovirus of low pathogenicity [1]. ALV-J was first isolated from meat-type chickens in 1988, primarily causing myeloid leukosis in chickens [2]. Although ALV-J has been eradicated from most countries, it continues to induce diseases in poultry populations in China. Co-infections of ALV-A, ALV-B, and ALV-J can occur in the same chicken, including commercial laying hens [3,4]. Such co-infections provide a potential opportunity for recombination between different subgroups of ALVs [5,6]. As a pathogen that is predominantly transmitted vertically through the eggs, the control of ALV in poultry populations is mainly achieved through eradication programs where the efficient and accurate detection and removal of carrier birds are crucial to prevent ALV transmission in the flocks. Successful ALV eradication is thus very much dependent on the efficacy of specific, sensitive, and reliable diagnostic methods for detecting ALV-positive carrier birds.

In the past two decades, a number of routine ALV detection methods including enzyme-linked immunosorbent assay (ELISA), immunofluorescence assay (IFA), and different types of PCR together with virus isolation protocols have been widely used in ALV eradication programs. However, these methods have advantages as well as drawbacks. For example, the widely used group-specific antigen detection ELISA cannot differentiate between exogenous and endogenous ALV as it detects the conserved p27 antigen [7]. While PCR and IFA can be used to detect and differentiate between endogenous and exogenous ALV, both techniques are not used widely in the field due to the requirement of sophisticated instrumentation [8,9]. Virus isolation in cell culture is used and considered as the gold standard diagnostic test. However, virus isolation is time-consuming, requiring at least 6 days to obtain the results, with additional time required for the identification of the ALV subgroups. Current PCR-related assays and isothermal methods such as RPA (recombinase polymerase amplification) and LAMP (loop-mediated isothermal amplification) also suffer from limitations including low specificity and the requirement of complex instrumentation [10,11].

**Figure 1:**
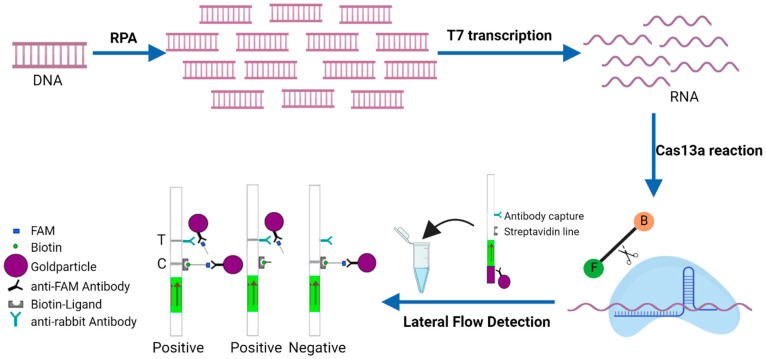
SHERLOCK detection of DNA from ALV-infected DF-1 cells. The region of interest is isothermally amplified by the RPA assay from DNA extracted from ALV-infected DF-1, then converted to RNA by T7 transcription. Cognate binding of the Cas13a–crRNA complex to amplified RNA targets triggers the collateral activity of Cas13a, which cleaves RNA reporters tagged with a fluorescein (F) and biotin (B) at each end. Detection is then performed as a colorimetric lateral flow reaction by placing the lateral flow strip into the reaction tube. The result can be visualized by the accumulation of the FAM/Biotin ssRNA reporter that conjugates to anti-FAM gold nanoparticles at the control or test lines depending on whether the reporter is intact. In the absence of reporter RNA cleavage, the RNA reporter is absorbed at the streptavidin line and captures anti-FAM antibodies. If the RNA reporter is destroyed by the collateral effect, then antibodies will flow through to a second capture line.

Recently, orthologs of CRISPR-associated enzymes such as Cas13a nuclease have shown great promise in nucleic acid detection assays in different biological systems, including many viruses [12,13]. Cas13a is an RNA-guided RNA nuclease that targets and cleaves the RNA. After Cas13a cleaves its target RNA, it adopts an enzymatically “active” state and binds and cleaves neighboring non-targeted RNA regardless of homology, which is referred to as “collateral cleavage”. It is this property of Cas13a that led to the establishment of SHERLOCK (specific high-sensitivity enzymatic reporter unlocking), a Cas13a-based molecular detection platform. SHERLOCK combines RPA (recombinase polymerase amplification) with the T7 transcription of amplified DNA to RNA and the collateral effect of CRISPR-Cas13a. The assay starts with pre-amplification of either a DNA (RPA) or RNA (RT-RPA) target input, followed by the conversion of amplified DNA to RNA via T7 transcription and then detection by Cas13−crRNA complexes, which activate and cleave RNA reporters that are used to create a signal after being cleaved if the target sequence is present in the pool of amplified nucleotides. Detection can be performed as a colorimetric lateral flow reaction with the RNA reporter flanked by a fluorescein and biotin on separate ends or a fluorescence-based reaction with the reporter sequence coupled to a fluorophore on one end and a quencher on the other end [14]. While the performance of RPA-based assays is variable and influenced by multiple factors including primers, template sequence, and amplicon length, SHERLOCK is fairly robust. In combination with lateral flow RNA reporters or quenched fluorescent RNAs, SHERLOCK can generate a colorimetric lateral flow or fluorescent readout, respectively, upon Cas13a recognition of target nucleic acid species with high specificity at single-nucleotide level discrimination and single-molecule sensitivity. SHERLOCK has been successfully applied for the detection of multiple viruses including Avian influenza A (H7N9) virus, Dengue virus (DENV), Zika virus (ZIKV), Ebola virus, SARS-CoV-2, Feline calicivirus (FCV), Porcine reproductive, and respiratory syndrome virus (PRRSV), as well as other molecules such as N1-methyladenosine and miRNA [15,16,17,18,19,20,21]. In the present study, the Cas13a detection using the purified Leptotrichia wadei Cas13a (LwaCas13a), a custom FAM-Biotin reporter and in vitro transcribed target RNA corresponding to the viral glycoprotein gp85 gene with a lateral flow readout, was developed for the visual detection of proviral DNA extracted from ALV-A-, ALV-B-, and ALV-J-infected DF-1 cells (Figure 1). The assay has proved to be sensitive and specific.

## 2. Materials and Methods

### 2.1. Viruses and Nucleic Acid Extraction

Virus stocks of prototype ALV subgroups ALV-A (RAV-1), ALV-B (RAV-2), ALV-C (RAV-49), ALV-D (RAV-50), ALV-E (RAV-0), and ALV-J (HPRS-103) maintained in the Viral Oncogenesis group at the Pirbright Institute were used in the experiments. ALV stocks were inoculated into DF-1 cells cultured in T25 flasks with Dulbecco’s modified Eagle’s medium (DMEM) (Invitrogen, Waltham, MA, USA) supplemented with 10% fetal bovine serum (FBS) at 38.5 °C in a 5% CO_2_ incubator. Passaging of the infected DF-1 cells was carried out two times in DMEM containing 2% FBS at 38.5 °C in a 5% CO_2_ incubator for 5 days. The harvested cells were lysed in 1× Proteinase K-based DNA extraction buffer (10 mM Tris-HCl, pH 8, 1 mM EDTA, 25 mM NaCl, and 200 µg/mL Proteinase K) at 65 °C for 30 min for the extraction of ALV subgroup-specific proviral DNA for RPA amplifications. DNA extracted from REV- and MDV-transformed cell lines was used as controls to determine the specificity of the assay.

### 2.2. RPA Primer Design and crRNA Preparation

For designing the RPA primer sets (between 30 and 35 nt), the previously published ALV sequences from NCBI were first aligned using DNASTAR. Based on the results of the sequence comparison, specific primers mainly targeting the gp85 gene were designed for discrimination of the three subgroups with amplicon sizes between 100 and 200 nt. The T7 promoter sequence was appended to the 5’end of the RPA forward primers. Primers were synthesized by Integrated DNA Technologies (IDT). To generate a complete crRNA template, the 28 nt spacer sequence was joined with a 5′direct repeat (DR) sequence with an additional upstream T7 RNA polymerase promoter sequence (T7-3G) to allow for T7 transcription [14]. The entire sequence was synthesized as the DNA reverse complement and the crRNAs were generated through in vitro transcription (IVT) according to the instructions of the HiScribe T7 Quick High Yield RNA Synthesis Kit (New England Biolabs, Ipswich, MA, USA). Briefly, the crRNA templates and T7-3G oligonucleotides were annealed after a 5 min denaturation at 95 °C in a PCR thermocycler and slow cooling to 4 °C. Double-stranded DNA was then transcribed to crRNA by incubation for 4 h at 37 °C and purified using Agencourt RNA Clean XP Kit (Beckman Coulter, Brea, CA, USA) according to the manufacturer’s instructions.

### 2.3. Expression and Purification of LwaCas13a

LwaCas13a protein expression and purification were carried out as previously reported with modifications [14]. Rosetta (DE3) competent cells transformed with the pC013-TwinStrep-SUMO-huLwaCas13a plasmid (https://www.addgene.org/90097/, accessed on 5 October 2019) were grown up and 20 mL of overnight culture was added into two liters of Luria Broth (LB) medium and incubated in a shaking incubator at 37 °C. When the OD600 value of the culture reached 0.6, protein expression was induced using IPTG to a final concentration of 500 μM and the cells were grown at 25 °C for 16 h. Cell pellets obtained after centrifugation at 3000× *g* for 20 min at 4 °C were crushed and resuspended in lysis buffer (250 mM NaCl, 20 mM Tris-HCl, 1 mM DTT, and pH 8.0). The suspension was sonicated on ice followed by centrifugation. The supernatant containing the protein was incubated with 5 mL of Strep-Tactin Sepharose (GE Healthcare, Chicago, IL, USA) for 2 h at 4 °C by gentle shaking and protein-bound Strep-Tactin resin was washed three times with cold lysis buffer. The resin was then resuspended in SUMO digest buffer (30 mM Tris-HCl, 500 mM NaCl, 1 mM DTT, 0.15% NP-40, and pH 8.0), along with 250 U of SUMO protease to cleave overnight at 4 °C with gentle rotation. To maximize protein elution, the resin was washed twice with one column volume of lysis buffer following the separation of the resin from the suspension by gravity flow. For cation exchange and gel filtration purification, 250 mM of elute diluted in cation exchange buffer (5% glycerol, 1 mM DTT, 20 mM HEPES, and pH 7.0) was loaded into a 5 mL HiTrap SP HP cation exchange column (GE Healthcare) via fast protein liquid chromatography (FPLC; AKTA PURE, GE Healthcare). The protein was then eluted over a salt gradient with 250 mM to 2 M NaCl in the elution buffer/cation exchange buffer. The presence of recombinant protein in the resulting fractions was tested by SDS-PAGE. The protein-containing fractions were pooled and concentrated to 1 mL in S200 size-exclusion buffer (1 M NaCl, 10 mM HEPES, 2 mM DTT, 5 mM MgCl2, and pH 7.0) using a centrifugal filter unit (Millipore, Burlington, MA, USA). The concentrated protein was then loaded onto a gel filtration column (Superdex 200 Increase 10/300 GL; GE Healthcare) for FPLC. Fractions containing protein were pooled and the buffer was exchanged with storage buffer (50 mM Tris-HCl, 600 mM NaCl, 5% glycerol, and 2 mM DTT) and stored at −80 °C. 

### 2.4. LwaCas13a Detection Assay

Detection reactions, consisting of RPA pre-amplification and T7 transcription for the detection of Cas13a function, were performed as described in Max et al. [14] with a few modifications. An amount of 3 µL of 10 µM forward primer, 3 µL of 10 µM reverse primer, 30 µL TwistAmp Rehydration Buffer, and 6 µL nuclease-free water were added to the RPA strips containing a dried enzyme pellet to prepare four individual RPA reactions that can be scaled up according to the reactions needed, followed by adding 1 µL of DNA template extracted from infected DF-1 cells to each reaction. Reactions were allowed to proceed for between 10 min and 40 min at 37 °C according to the instructions of the TwistAmp^®^ Basic kit (TwistDx, Maidenhead, UK). To establish the Cas13a lateral flow detection with a visual readout, 10 μL of LwaCas13a assay reaction consisting of 1.0 µL RPA product, 0.25 µL HEPES (1M, pH 6.8), 0.1 µL MgCl_2_ (1M), 0.4 µL rNTP solution mix (25 mM each), 1.0 µL LwaCas13a (63.3 µg/mL), 0.5 µL Murine RNase inhibitor (40 U/µL), 0.25 µL T7 RNA polymerase (5 U/µL), 0.5 µL crRNA (10 ng/µL), 0.1 µL LF-RNA reporter 1 (100 µM), and 6.25 µL nuclease-free water was first prepared. Following the addition of 50 μL HybriDetect assay buffer, the HybriDetect 1 lateral flow strip (TwistDx) was dipped into the solution and incubated for 3 min for the readout. After incubation, the images were taken using a smartphone.

### 2.5. Specificity and Sensitivity of the Assay

DNA samples containing the proviral DNA of the different ALV subgroups extracted from DF-1 cells infected with the prototype strains, together with REV and MDV as negative controls, were used to determine the specificity of the assay.

In order to determine the sensitivity of the assay, gel-extracted PCR amplicons of ALV-A/B/J amplified by specific primers targeting the *gp85* region (Table 1) were used for cloning into a plasmid as standard references. Concentrations of all three standard references were determined by UV spectrophotometry. DNA copy numbers (copies/μL) were calculated using the formula (6.02 × 10^23^) × (ng/μL × 10^−9^)/(DNA length × 660). An amount of 1 μL of each serial dilution containing 10^4^ to 10^1^ copies/μL DNA was used as the input DNA template to detect the sensitivity of this method.

### 2.6. Application of the Assay on Field Samples

In order to verify the application of the LwaCas13a assay for diagnostic purposes on field samples, 23 validated ALV-A/B/J-positive hepatic tissue samples collected from different chicken farms were used for the extraction of DNA and RNA by using a AxyPrep^TM^ Body Fluid Viral DNA/RNA Miniprep Kit (AXYGEN, 20019KC5, New York, USA). An amount of 1 μL of DNA extracted from each specimen was used as a template in the PCR and RPA reactions, respectively. The presence of ALV-A/B/J proviral DNA was confirmed with subgroup-specific primers targeting the *gp85* gene (Table 1) using the following cycle conditions: pre-denaturation at 95 °C for 5 min; 30 cycles of denaturation at 95 °C for 20 s, annealing at 58 °C for 30 s, extending at 72 °C for 1 min. Samples were assayed and analyzed following the previously described Cas13a detection and lateral flow methods. The images were taken using a smartphone.

## 3. Results

### 3.1. Design of Primers and crRNAs and crRNA Preparation 

As described above, the assay starts with pre-amplification of either a DNA (RPA) or RNA (RT-RPA) target input. For RPA, primers are typically designed with NCBI Primer-BLAST to be 30~35 nt and the amplicon sizes are 100~200 bp. In order to target a broad range of ALV strains in the same subgroup, the conserved crRNA sequences were identified by the alignment of multiple *gp85* gene sequences within each ALV subgroup retrieved from the NCBI. To identify the best primers and crRNA, we designed one crRNA with six pairs of RPA primers for ALV-A, one crRNA with three pairs of RPA primers for ALV-B, and two crRNAs for ALV-J, one with three pairs and the other one with two pairs of RPA primers, respectively. The sequences of the designed RPA primers and crRNA IVT templates are listed in Table 2. Each of the ALV subgroup-specific crRNAs were generated by IVT from DNA templates, and 5 µL per aliquot (300 ng/µL concentration in nuclease-free water) was stored at −80 °C.

### 3.2. Expression and Purification of LwaCas13a

**Figure 2:**
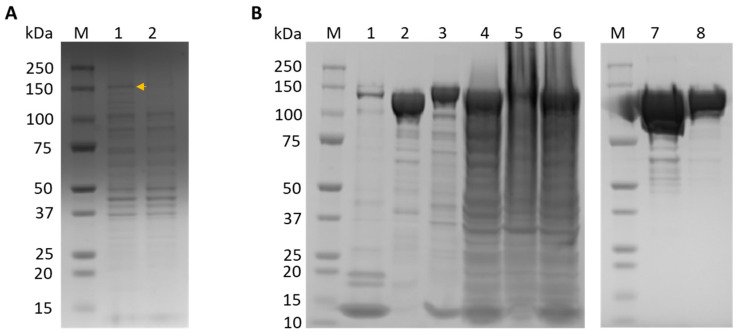
Expression and purification of LwaCas13a. (**A**) LwaCas13a expression detection. Lane M, protein marker; lane 1, IPTG-induced bacterial sample (the LwaCas13a band is indicated by the arrow); lane 2, uninduced bacterial sample. (**B**) LwaCas13a purification. Lane M, protein marker; lane 1, Strep-Tactin resin post SUMO cleavage; lane 2, eluted fraction post SUMO protease cleavage; lane 3, Strep-Tactin resin before SUMO protease cleavage; lane 4, flow-through following Strep-Tactin batch binding; lane 5, cell pellet after clearing of lysate; lane 6, cleared cell lysate; lane 7, final product after SEC; lane 8, concentrated sample post IEC.

Rosetta *E. coli* cells transformed with TwinStrep-SUMO-LwaCas13a protein expression plasmid were induced with 500 μM IPTG and grown for 16 h at 25 °C. The harvested cells were resuspended, sonicated, and centrifuged to remove the debris. After IPTG induction, the LwaCas13a protein was expressed (Figure 2A), and the target protein was mainly in the supernatant. The recombinant protein was enriched from the total cell protein by affinity Strep-Tactin purification. The native LwaCas13a was obtained following removal of the SUMO tag after digestion with SUMO protease and further purified using ion-exchange (IEC) and size-exclusion chromatography (SEC) on an FPLC system. The expression and purification of the LwaCas13a protein was determined by SDS-PAGE and Coomassie blue staining as shown in Figure 2. The purified 138.5 kD LwaCas13a was then diluted to a final concentration of 2 mg/mL in protein storage buffer and stored as 5 µL aliquots at −80 °C.

### 3.3. Validation of the LwaCas13a Lateral Flow Detection

Lateral flow strips from TwistDx are designed to detect biotin and FAM-labeled RNA reporter. As shown in Figure 1, the first line (control line) with streptavidin will bind to biotin, capturing all the intact probes. Anti-fluorescein antibodies labeled with gold nanoparticles (NPs) will bind the fluorescein end of the reporter and form a dark purple color at this first line. When RNA reporters are cleaved because of target presence and collateral activity, gold NP–labeled antibodies will flow over to a second line of anti-rabbit secondary antibody (test line), capturing all the antibodies and forming a dark purple color at the second line that indicates the presence of the target. As a result, the negative sample only forms one band at the control line as all reporter molecules are intact and captured at this line. As shown and described by others, a faint band may appear at the test line for the non-target control or the negative strips are allowed to sit at room temperature for over 10 min, but this signal is much fainter than a true positive signal [22,23,24]. For the positive samples, there could be one test line present when the RNA reporter is fully cleaved or both a control and test line present if the RNA reporter is partially cleaved. 

**Figure 3:**
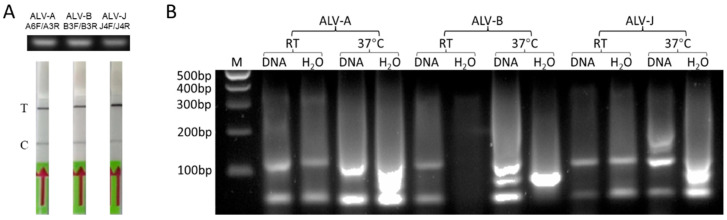
Validation of the LwaCas13a lateral flow detection. (**A**) Top panel: agarose gel electrophoresis of RPA products using specific primers A6F/A3R for ALV-A, B3F/B3R for ALV-B, and J4F/J4R for ALV-J. Bottom panel: the corresponding Cas13a assay result of ALV/A/B/J by imaging of the lateral flow dipstick is displayed. T: test line, C: control line. (**B**) RPA pre-amplifications optimization. RPA reactions were run for ALV-A/B/J at room temperature (RT) or 37 °C with proviral DNA and water control with primer pairs A6F/A3R, B3F/B3R, and J4F/J4R for subgroups ALV-A/B/J, respectively. Proviral DNAs of RAV-1, RAV-2, and HPRS-103 extracted from infected DF-1 were used as templates for ALV-A, ALV-B, and ALV-J, respectively.

**Figure 4:**
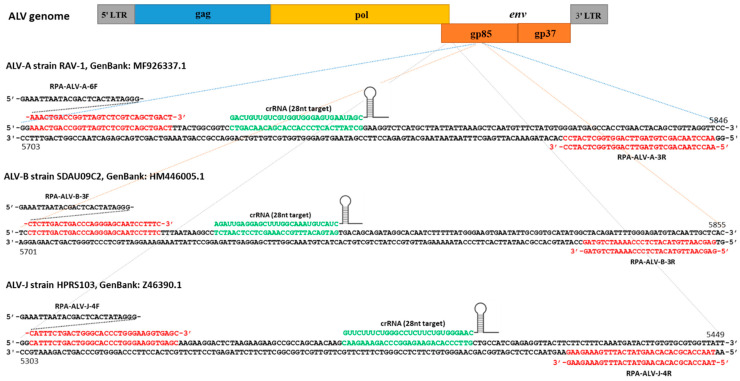
Binding sites of RPA primers and crRNAs. The RPA primers and crRNA sequences targeting *gp85* of the ALV-A/B/J genome selected through experimental optimization are shown. The nucleotide sequences and the numbering showing the genomic location at both ends of the selected sequences are based on RAV-1 (GenBank accession number: MF926337.1), SDAU09C2 (GenBank accession number: HM446005.1), and HPRS103 (GenBank accession number: Z46390.1) for ALV-A, ALV-B, and ALV-J, respectively.

Optimal RPA was performed for different amplification durations ranging from 20 to 60 min. Each reaction includes 9 µL of the reconstituted RPA mixture and 1 µL of tested sample in a PCR strip tube. In RPA pre-amplification, the negative control with the target molecule known to be absent was also included. The assay using the DNA extracted from prototype strains of ALV-A-, B-, and J- (RAV-1, RAV-2, and HPRS-103, respectively)-infected DF-1 cells showed that A6F/A3R, B3F/B3R, and J4F/J4R primer sets were efficient and capable of specific amplification producing the expected test bands (Figure 3A, top panel). The binding sites of these RPA primers and crRNAs which are used in the subsequent LwaCas13a lateral flow detection are shown in Figure 4. To optimize the reaction conditions, different temperatures and incubation times were examined. While the test bands that correlated to 10 min of RPA were weak, extending the amplification time to 30 min and longer increased the intensity of the test band. Based on the optimization data, the amplification duration was set at 40 min for ALV-A and 30 min for ALV-B/J RPA reactions. Since the RPA amplification reaction is initiated following MgOAc addition, we also want to know if the nucleic acid amplification was initiated during sample preparation at room temperature. We prepared two pairs of RPA reactions consisting of one positive and one negative for each pair and allowed one pair to be at room temperature and the other pair to be at 37 °C for 30 min. The crude RPA products were examined on a 2% agarose gel. As shown in Figure 3B, no significant changes between positive and negative samples of ALV-A and J were observed on the gel at room temperature amplification. However, the detection of the amplicons at 37 °C indicated apparent differences. 

Having obtained the specific RPA bands for the ALV-A, B, and J subgroups, the Cas13a assay was performed in a total volume of 10 μL. For this, Cas13a-SHERLOCK master mix was prepared by adding the components as described in Section 2. Subsequently, 50 μL HybriDetect assay buffer was added to the 10 μL Cas13a detection products followed by placing a HybriDetect 1 lateral flow strip (TwistDx) into the solution and incubation in an upright position. After 3 min incubation, the images of dipsticks were taken using a smartphone. As shown in Figure 3A, bottom panel, a strong band appeared in the test line region for all three samples tested, demonstrating the successful establishment of the Cas13a lateral flow detection of proviral DNA for the ALV-A/B/J subgroups. 

### 3.4. Specificity and Sensitivity of the Cas13a Lateral Flow Detection 

**Figure 5:**
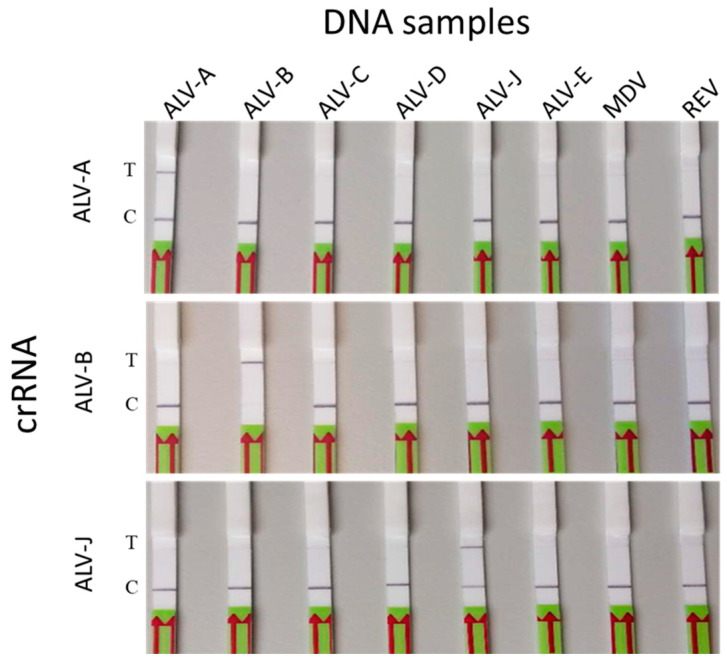
Specificity of ALV-A/B/J-LwaCas13a detection. Specificity detections of ALV-A/B/J with RPA at 37 °C for 30 min and Cas13a reaction at 37 °C for 40 min. DNA samples include proviral DNAs of RAV-1, RAV-2, RAV-49, RAV-50, HPRS-103, and RAV-60 for ALV-A, ALV-B, ALV-C, ALV-D, ALV-J, and ALV-E, respectively, and genomic DNAs of MDV (vaccine strain CVI-988) and REV infection cells. T: test line; C: control line.

**Figure 6:**
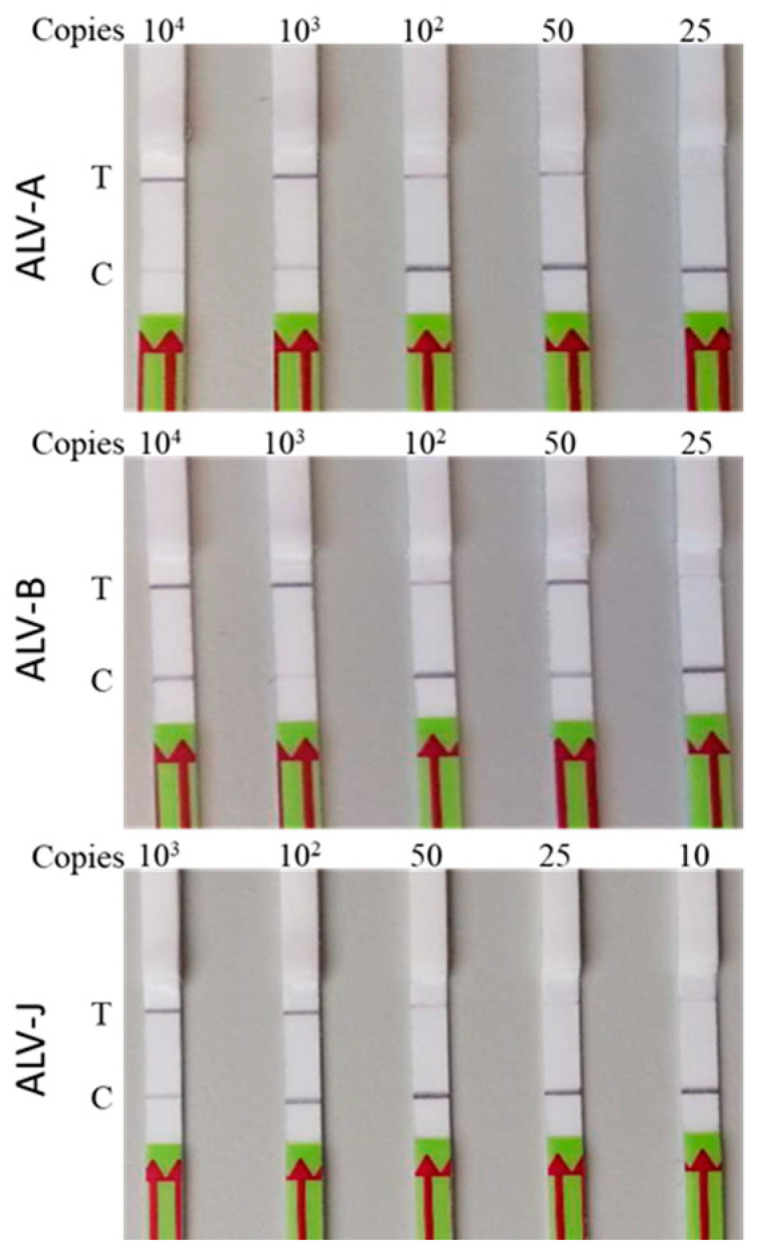
Sensitivity testing of ALV-A/B/J-LwaCas13a detection. Sensitivity detection results of ALV-A/B/J-Cas13a with RPA at 37 °C for 30 min and LwaCas13a reaction at 37 °C for 40 min. Gradient-diluted cloning vector of ALV-A/B/J plasmid DNAs were detected using ALV-A/B/J-LwaCas13a assays. The detection limit of ALV-A/B/J is 50 copies/reaction for all three. T, test line; C, control line.

The specificity of the assay for the detection of ALV-A, B, and J subgroups was determined by examining the signals for ALV subgroups C, D, and E and other avian oncogenic viruses REV and MDV. Results of the specificity test clearly demonstrated that the assay was ALV A, B, and J subgroup-specific with no cross reactivity (Figure 5). In order to determine the sensitivity of the assay, 10-fold serial dilutions containing 10^4^ to 10^2^ copies/μL followed by two 2-fold dilutions to make 50 and 25 copies/μL (an additional dilution to 10 copies/uL was made to ALV-J). Each of the individual DNA templates were tested as shown in Figure 6. Results of the sensitivity test demonstrated that each assay could reliably detect a minimum of approximately 50 copies of the respective DNA targets in each reaction.

### 3.5. Assay Performance on Field Samples

**Figure 7:**
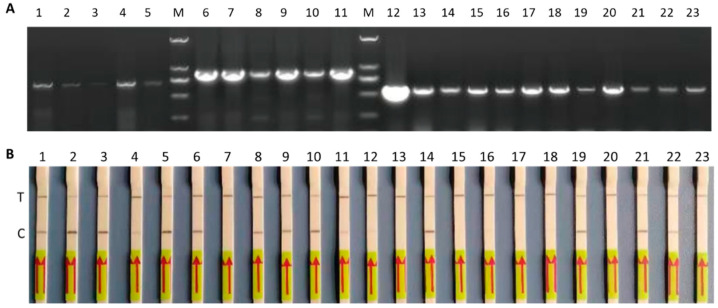
LwaCas13a detection on field samples. (**A**) Agarose gel electrophoresis of PCR products using specific primers. Lane M, DNA Marker 2000; lanes 1 to 5 are ALV-A-positive samples, lanes 6 to 11 are ALV-B-positive samples, and lanes 12 to 23 are ALV-J-positive samples. (**B**) ALV/A/B/J-LwaCas13a assay results with primer pair A6F/A3R for ALV-A samples (lanes 1–5), B3F/B3R for ALV-B samples (lanes 6–11), and J4F/J4R for ALV-J samples (lanes 12–23). T: test line, C: control line.

Finally, the assay was evaluated for its efficacy for the detection of ALV-A-, B-, and J-specific DNA in field samples of naturally infected cases. For this, we used 5 ALV-A-positive, 6 ALV-B-positive, and 12 ALV-J-positive field samples to determine the efficacy of detection of specific DNA, in comparison to PCR tests. As shown in Figure 7, lateral flow detection strips of ALV-A, B, and J subgroups demonstrated clear positive bands, correlating with the PCR tests carried out on these field samples, confirming the use of the Cas13a lateral flow assay for the specific proviral DNA detection of ALV-A, B, and J subgroups from field samples.

## 4. Discussion

Avian leukosis viruses, the common naturally occurring avian retrovirus pathogens, are associated with neoplastic diseases and other production problems in chickens [1]. With no effective vaccine or medication available, control of ALV infection is achieved mainly through systematic eradication program consisting of the early detection and removal of virus-shedding birds to break the chain of transmission that occurs through the congenital route and contact infections [25]. Several methods for ALV detection have been established. While antigen–antibody reaction-based detection can be used in the field with minimal equipment, the method has relatively low sensitivity [7,26]. PCR and RT-qPCR, the highly sensitive nucleic acid-based detection assays, are not suitable for poorly equipped laboratories or field diagnosis due to the requirement of expensive equipment [8,9,26]. Considering the importance of the accurate and sensitive detection of virus-infected birds for successful eradication, the availability of rapid, accurate ALV diagnostic tools is key for the success of the eradication.

In this study, a LwaCas13a detection assay was developed and optimized to detect viruses belonging to the common pathogenic subgroups ALV-A, ALV-B, and ALV-J. Primer sets and crRNAs specific for these subgroups were designed based on the consensus from the alignment of the nucleotide sequences of the viral *gp85* gene published in GenBank. Only the most distinct region that differed between different subgroups, but was highly conserved within *gp85* sequences of the same subgroup, was used to design the specific RPA primers. While the sequences of ALV-J *gp85* differed greatly from those of the other ALV subgroups, it has close sequence identity to env-like sequences of the EAV family of endogenous avian retroviruses [27,28]. As the ancient EAV-HP retroviral elements are present in all gallus species, these could interfere with the specific amplification of the ALV-J *gp85* gene. Thus, the chosen primers need to selectively amplify only the region specific to the exogenous ALV-J sequences. The H5/H7 primer set has been used successfully for detection of ALV-J [29]. The forward primer H5, located on the 3’ region of the pol gene, was conserved across several ALV subgroups. The reverse primer H7, located on a well-conserved region of the ALV-J *gp85* gene, could distinguish ALV-J from other subgroups. In our study, the primer pairs J1, J2, and J3 were first designed mainly focusing on the region around the H7 primer binding sequences. Although the experiments generated RPA amplification products, the Cas13a detection assay could not distinguish ALV-J from other exogenous ALV proviral DNA, probably due to the interference with the EAV-HP sequences. Hence, for the ALV-J detection, we modified the assay using another forward (JF4) and reverse (JR4) primer pair located at the 3’end of the pol and at the 5’end of the *gp85*, respectively. Tests using this new primer pair showed a good specificity to ALV-J amplification. Thus, the primer sets A6F/A3R, B3F/B3R, and J4F/J4R for RPA with the corresponding crRNAs for LwaCas13a detection performed accurately for the detection of the respective ALV subgroups.

In order to evaluate the specificity of the LwaCas13a detection method, we extracted proviral DNA from DF-1 cells infected with prototypes of ALV subgroups A, B, C, D, E, and J. In addition, DNAs from cells infected with Marek’s disease virus and reticuloendotheliosis virus were extracted for use as controls. As expected, only the ALV-A/B/J subgroup-infected DNA samples could be detected with the corresponding RPA primers and crRNAs by this method, demonstrating the specificity of the test (Figure 5). The LwaCas13a detection assay also showed a detection limit of 50 copies per reaction for each of the subgroups. Perhaps due to this limited sensitivity of the assay, no significant difference in intensity was observed between the T and C lines in the lateral flow strip-based readout of the assay, when a number of reporter RNAs were cleaved. Despite this, we have demonstrated that the LwaCas13a lateral flow can be used for specific and sensitive proviral DNA detection of the major ALV subgroups A, B, and J. When the turnaround time was considered, the described method in this study requires 1.5 h of average assay time including 30 min of rapid DNA extraction, 30 min of RPA for DNA amplification, and 40 min of LwaCas13a detection.

Unlike PCR-based DNA amplification assays, there are no rules for the design of RPA primers other than the suggestion from the TwistDx website (www.twistdx.co.uk/rpa/). Besides genuine amplifications, RPA also allows for undesired primer interactions such as hairpins and primer dimer formation. Some of such artefacts will serve as templates for further recombination/extension events and enter an exponential amplification. Then, the detectable low-molecular-weight DNA consisting of a primer-derived sequence (“prime noise”) will be generated (Figure 3B). The sensitivity of amplification and speed of RPA are directly affected by the amplicon size due to the effects of primer noise. An amplicon length ideally is between 100 and 200 bp, which tends to have an improved product/noise ratio. At low temperatures, amplicon doubling times lengthen more rapidly than energy consumption rates, which can result in fuel “burn-out”. The observations of our study are consistent with the information given by the manufacturer that RPA amplification occurs over a broad range of temperatures (25 to 42 °C), making the assay robust against inadvertent temperature changes. It has also been reported that RPA is quite robust to mismatches even with 5~9 base pair mismatches in the primers [30]. However, this is not the case with the mismatches in the crRNA:target duplex, where even two or more mismatches can prevent the activation of Cas13a. As a result, SHERLOCK can easily distinguish the sequences of similar viruses. Furthermore, due to the additional specificity of the crRNA, it is not necessary to evaluate primer sets for specific amplicons using gel electrophoresis.

Although the efficiency of RPA is variable and influenced by factors such as primer and template sequence, the collateral cleavage activity of Cas13a can compensate for the low efficiency of RPA, making this assay achieve satisfactory results [31]. Moreover, as both reactions can occur at 37 °C, the method only demands simple heat source systems such as chemical heaters, water baths, or even body heat, and the results can be visualized with naked eye [16,20]. Furthermore, the method is not dependent on the cold chain as all the critical RPA reagents can be provided as a single lyophilized pellet. The entire procedure can be completed within 1.5 h and requires only a small sample volume of 10 µL for both RPA and SHERLOCK. Requiring only a basic heat source for incubation and a simple lateral flow readout, the LwaCas13a assay developed in this study can be considered as a rapid, efficient, and accurate field test for the diagnosis of ALV in low-resource settings.

## 5. Conclusions

A visual nucleic acid detection method based on CRISPR-LwaCas13a was established for the common pathogenic ALV subgroups A, B, and J. The method which combines RPA pre-amplification with the identification of a specific crRNA sequence makes the LwaCas13a lateral flow detection more accurate. The current study presented for the first time an alternative tool for the rapid, sensitive, and specific detection of ALV, particularly in resource-poor settings.

## Figures and Tables

**Table 1:** Specific primers for ALV-A/B/J identification.

Targets	Sequences (5′-3′)	Product Sizes (bp)
ALV-A	H5: GGATGAGGTGACTAAGAAAG	694
	envA: AGAGAAAGAGGGGCGTCTAAGGAGA	
ALV-B	H5: GGATGAGGTGACTAAGAAAG	846
	envB: ATGGACCAATTCTGACTCATT	
ALV-J	H5: GGATGAGGTGACTAAGAAAG	545
	H7: CGAACCAAAGGTAACACACG	

**Table 2:** The sequences of primers and crRNAs designed for Cas13a detection in this study.

Primer	Sequence (5′-3′)	Sizes
ALV-A1-F	GAAATTAATACGACTCACTATAGGGACTGGCGGTCCTGACAACAGCACCACCCTCACT	143 bp
ALV-A1-R	GTAATATTAGTAATGTTAGGGAGAGACTGGGAAC	
ALV-A2-F	GAAATTAATACGACTCACTATAGGGGATATGTCTCTGATACAAATTGCGCCACCT	170 bp
ALV-A2-R	CAGCTGTAGTTCAGGTGGCTCATCCCACATAG	
ALV-A3-F	GAAATTAATACGACTCACTATAGGGCGGAAACTGACCGGTTAGTCTCGTCAGCTGACT	147 bp
ALV-A3-R	AACCTAACAGCTGTAGTTCAGGTGGCTCATCC	
ALV-A4-F	GAAATTAATACGACTCACTATAGGGATATGTCTCTGATACAAATTGCGCCACCTCGGA	176 bp
ALV-A4-R	AACCTAACAGCTGTAGTTCAGGTGGCTCATCCCA	
ALV-A5-F	GAAATTAATACGACTCACTATAGGGCTGATACAAATTGCGCCACCTCGGAAACTGAC	165 bp
ALV-A5-R	CTAACAGCTGTAGTTCAGGTGGCTCATCCCA	
ALV-A6-F	GAAATTAATACGACTCACTATAGGGAAACTGACCGGTTAGTCTCGTCAGCTGACT	165 bp
ALV-A6-R	TAATGTTAGGGAGAGACTGGGAACCTAACAG	
crRNA IVT template for A1-A6	CTGACAACAGCACCACCCTCACTTATCGGTTTTAGTCCCCTTCGTTTTTGGGGTAGTCTAAATCCCCTATAGTGAGTCGTATTAATTTC	
ALV-B1-F	GAAATTAATACGACTCACTATAGGGCTAATATTACTCAGATCCCTAGTGTGGCTGG	134 bp
ALV-B1-R	AGGATTGCTCCCTGGGTCAGTCAAGAGGATG	
ALV-B2-F	GAAATTAATACGACTCACTATAGGGTGGGACCGGAGACAAGTTACACACATCCTCTTG	157 bp
ALV-B2-R	TGTAGCCATATGCACCGCAATATTCACTTCCCAT	
ALV-B3-F	GAAATTAATACGACTCACTATAGGGCTCTTGACTGACCCAGGGAGCAATCCTTTC	155 bp
ALV-B3-R	GAGCAATTGTACATCTCCCAAAATCTGTAG	
crRNA IVT template for B1-B3	TCTAACTCCTCGAAACCGTTTACAGTAGGTTTTAGTCCCCTTCGTTTTTGGGGTAGTCTAAATCCCCTATAGTGAGTCGTATTAATTTC	
ALV-J1-F	GAAATTAATACGACTCACTATAGGGGATATTTTAGGGTCTCAGATGATCAAGAACGGAAC	142 bp
ALV-J1-R	CTTCCACCCCACCAGTCCCATTAAAATTCCCATCA	
ALV-J2-F	GAAATTAATACGACTCACTATAGGGCTTGATAAAGGCTCTTAACACAAACCTCCCTTG	191 bp
ALV-J2-R	CTTCCACCCCACCAGTCCCATTAAAATTCCCATCA	
ALV-J3-F	GAAATTAATACGACTCACTATAGGGGTACGTGTGTTACCTTTGGTTCGATGTGCT	172 bp
ALV-J3-R	GATTGGTTGACATAGGGTCTTATACGAGGGTC	
crRNA IVT template for J1-J3	GCTATAAAGAGAACAATCACAGCAGAGTGTTTTAGTCCCCTTCGTTTTTGGGGTAGTCTAAATCC CCTATAGTGAGTCGTATTAATTC	
ALV-J4-F	GAAATTAATACGACTCACTATAGGGCATTTCTGACTGGGCACCCTGGGAAGGTGAGC	147 bp
ALV-J4-R	TAACCACGCACACAAGTATCATTTGAAAGAAG	
ALV-J5-F	GAAATTAATACGACTCACTATAGGGGGATGAGGTGACTAAGAAAGATGAGGCGAGC	183 bp
ALV-J5-R	ACACAAGTATCATTTGAAAGAAGAAGTAACC	
crRNA IVT template for J4-J5	CAAGAAAGACCCGGAGAAGACACCCTTGGTTTTAGTCCCCTTCGTTTTTGGGGTAGTCTAAATCCCCTATAGTGAGTCGTATTAATTTC	

## Data Availability

All datasets generated for this study are included in the article.
